# Platelet Indices as Novel Surrogate Markers for the Prognosis of COVID-19 Infection: An Observational Study

**DOI:** 10.7759/cureus.62243

**Published:** 2024-06-12

**Authors:** Vivek Lahane, Sourya Acharya, Samarth Shukla, Sunil Kumar, Kashish Khurana, Sarang S Raut, Ajinkya Kadu

**Affiliations:** 1 Department of Medicine, Jawaharlal Nehru Medical College, Datta Meghe Institute of Higher Education and Research, Wardha, IND; 2 Department of Pathology, Jawaharlal Nehru Medical College, Datta Meghe Institute of Higher Education and Research, Wardha, IND

**Keywords:** ards, rt-pcr, platelets indices, infection, covid-19

## Abstract

Background

The new severe acute respiratory syndromecoronavirus 2 (SARS-CoV-2) causes severe acute respiratory illness accountable for causing the coronavirus disease 2019 (COVID-19) illness. Thrombotic issues, acute respiratory distress syndrome (ARDS), and cytokine storm are significant contributors to morbidity and mortality in patients with COVID-19. Elevated D-dimer levels and prothrombin times are further indicators of abnormal coagulation parameters in COVID-19 patients. This study aimed to study the platelet indices as prognostic markers in COVID-19 infection.

Methods

In this prospective observational study, 150 real-time reverse transcription-polymerase chain reaction (RT-PCR)-positive COVID-19 patients were enrolled between October 2020 and September 2021. All the subjects were screened and explained the study procedure in their native language. Following enrolment, a detailed history and physical examination were performed. Subsequently, laboratory investigations were performed, and patients were subjected to high-resolution computed tomography (HRCT) examination to classify patients into mild, moderate, and severe according to the severity of the illness. The platelet indices taken into account were plateletcrit (PCT) in percentage, platelet count (PLT) in lakh per microlitre, mean platelet volume (MPV) in femtolitres, and platelet distribution width (PDW) in femtolitres.

Results

The mean PLT was significantly greater among survivors than non-survivors (2.03 ± 0.72 versus 1.76 ± 0.47; p-value = 0.018). The mean MPV (10.42 ± 0.53 versus 9.22 ± 0.64; p-value <0.0001) and PDW (17.99 ± 1.53 versus 16.54 ± 0.91 fl; p-value <0.0001) were significantly greater among non-survivors than survivors. However, the mean PCT was significantly greater among survivors than non-survivors (0.22 ± 0.03% versus 0.18 ± 0.33%; p-value <0.0001). At a cut-off of 0.213, the sensitivity and specificity of PCT in predicting death were found to be 79.2% and 74.5%, respectively. At a cut-off of 16.75, the sensitivity and specificity of PDW in predicting death were found to be 68.8% and 59.8%, respectively.

The findings demonstrated a relationship between elevated MPV and PDW and mortality and severe COVID-19 infection. Increased PCT was connected to higher survival, with a specificity and sensitivity of 87.5% and 75.5%, respectively, and MPV >9.75 may predict death. PDW >16.75 exhibited a specificity and sensitivity of 68.8% and 59.8%, respectively, in predicting death. With comparable sensitivity and specificity of 79.2% and 74.5%, PCT >0.213 may predict death.

Conclusion

In severely sick COVID-19 patients, platelet indices should be routinely calculated and can be utilized as simple, low-cost prognostic indicators.

## Introduction

Infection with the coronavirus disease 2019 (COVID-19) illness initially emerged in Wuhan's seafood Chinese province of Hubei wholesale market. The novel severe acute respiratory syndrome coronavirus 2 (SARS-CoV-2) is to blame [1]. SARS-CoV-2 spread rapidly over the planet, necessitating the World Health Organization (WHO) to classify it as a global pandemic [2].

COVID-19 infection can be a significant reason for the hospitalization and death of individuals with COVID-19 illness with significant contributors such as thrombotic problems, airway obstruction disorder with the acute episode (acute respiratory distress syndrome (ARDS)), and inflammatory storm [3,4]. In the available data, it is appraised that COVID-19 causes haematological changes with some dissimilarities in severe and non-severe cases. The commonest haematological findings are leukopenia, lymphopenia, and thrombocytopenia [5].

Platelets have an important and vital role in haemostasis, coagulation, and thrombosis. Moreover, they also add to immunity and inflammatory changes. The platelet-related indices available are the plateletcrit (PCT), platelet count (PLT), platelet-large cell ratio (P-LCR), mean platelet volume (MPV), and platelet distribution width (PDW) [6]. There occur alterations in platelet function (apoptosis) due to the interplay between pathogens and platelet causes [7].

Platelets are induced by cytokines and have a significant place in leukocyte migration and endothelium binding due to the production of pro-inflammatory cytokines, which contribute to inflammation. Size-wise, larger platelets are hyper-reactive, producing larger quantities of thromboxane A2 and cytokines, whose granule contents are dense as compared to smaller platelets leading to increased requirements during the acute inflammatory stages [8]. Hence, platelet indices change in an inflammatory state. The larger development of reactant molecules in the acute phase and cytokines significantly alters megakaryopoiesis leading to platelet indices being altered by the release of tiny volumes of platelets from the bone marrow [9].

The release of excessive levels of transforming growth factor (TGF), interferon (IFN), interleukin (IL)-1b, IL-6, IL-12, IL-18, and IL-33 is associated with COVID-19 causing changes in platelet indices, according to evidence in the literature [10-12]. Specifically, it has been demonstrated that COVID-19 has considerable impacts on haemostasis and the haematological system [13]. According to research, COVID-19 sufferers are at risk of thrombotic events and hypercoagulation. In COVID-19 individuals, aberrant coagulation parameters are also indicated by activated partial thromboplastin times and higher D-dimer concentrations [14]. The results of the research have shown the necessity for an in-depth study into how platelet indices and PLT affect the physiopathology and the outlook of the disease [15,16]. Platelet indices can be a straightforward predictive and diagnostic tool in COVID-19 based on these findings. Thus, with COVID-19 infection, we had tried to evaluate platelet indices as a prognostic marker in terms of recovery, severity, and death in infected patients.

## Materials and methods

This prospective and observational study was conducted in the Department of Medicine, Acharya Vinoba Bhave Rural Hospital, a tertiary care teaching hospital situated in the rural area of Wardha District. The study spanned over a two-year period, from October 2020 to September 2021. The study enrolled 150 real-time reverse transcription-polymerase chain reaction (RT-PCR)-positive COVID-19 patients after conforming to the ethical guidelines and receiving institutional ethics committee certification. Approval for the study was obtained from Datta Meghe Institute of Medical Sciences (DMIMS) Institutional Ethics Committee (approval number: DMIMS(DU)/IEC/2020-21/9299).

All patients aged 18 or more years, patients of either gender, and RT-PCR-positive COVID-19 patients were included in the study. In contrast, exclusion criteria were patients with hypersplenism, patients with cirrhosis or liver disease, patients with immune thrombocytopenic purpura, patients with bone marrow disorder, patients with other acute infections, and patients receiving drugs that affect platelet function or blood coagulation.

At the time of data collection, the following parameters were noted in all the patients: age, sex, and history of comorbidities such as diabetes, hypertension, and asthma. History of smoking habits and alcohol consumption were also noted. A chest high-resolution computed tomography (HRCT) scan was performed to assess the presence of COVID-19-related pulmonary lesion. The CT severity score (CTSS) was determined at enrolment. On the basis of the involvement of each five lung zones, the CTSS was calculated as follows: no lobar involvement, score of 0; less than 5%, score of 1; 5-25%, score of 2; 26-50%, score of 3; 51-75%, score of 4; and more than 75%, score of 5. Some of the individual lobar scores indicated the overall severity of the five lobes, that is, a 0 score indicates normal severity, less than 7 indicates mild severity, 8-17 indicates moderate severity, and more than 17 indicated severe severity.

A complete blood count was done after withdrawing samples aseptically at the time of admission. The sample was collected in a dipotassium ethylenediaminetetraacetic acid (EDTA) bulb and tested within one hour when maintained at room temperature. An automated cell counter, the Beckman Coulter Unicel DxH 800 Hematology Analyzer (Beckman Coulter, Inc., Brea, CA, USA), was used, which provided the values of hemoglobin, total PLT, and total leukocyte count.

Platelet indices were obtained from the complete blood count Coulter report. MPV is the measure of the size of the platelets in the blood. The normal range of MPV was 7.2-11.7 fl. PCT is the volume occupied by platelets in the blood as a percentage and calculated according to the formula PCT = PC × MPV/10,000. The normal range for PCT was 0.22-0.24%. PDW is a measurement of platelet anisocytosis calculated from the distribution of individual platelet volumes, having a reference range of 10.0-17.9%. The calculated number of platelets in a volume of blood is usually expressed as platelets per cubic millimeter (mm^3^) of whole blood. The normal range of PLT was 150,000-450,000 platelets per microliter of blood [12,13]. Other parameters were serum creatinine and serum electrolyte (sodium and potassium) indicators of inflammation like IL-6, lactate dehydrogenase (LDH), ferritin, D-dimer, and C-reactive protein (CRP). The investigation included 150 COVID-19 sufferers with positive RT-PCR outcomes. Each participant underwent screening, and the study's methodology was described to them in their own language. The study only included participants who agreed to participate and gave their written consent. From the patient's history and clinical examination, a COVID-19 patient was first recognized. To validate the COVID-19 diagnosis, nasopharyngeal and oropharyngeal swabs were taken for RT-PCR. Subsequently, patients were subjected to CT examination to classify patients according to disease severity, i.e., mild, moderate, and severe. Finally, the outcome was measured in terms of recovery, severity, and death in infected patients.

Statistical analysis

Data was collected and graphics were designed by Microsoft Office Excel 2019. The final analysis was done with the use of the IBM SPSS Statistics for Windows, V. 23.0 (IBM Corp., Armonk, NY, USA). The categorical and continuous variables are represented as frequency (percentage) and mean (standard deviation (SD)), respectively. The chi-squared test and independent samples t-test were used to assess the association between various categorical and continuous variables, respectively. One-way ANOVA was used to assess the association between platelet indices and disease severity. Receiver operating characteristic (ROC) analysis was performed to determine the best cut-off point of platelet indices in predicting COVID-19-related mortality. Multiple regression analysis was performed for platelet indices. A two-tailed probability value of <0.05 was considered statistically significant.

## Results

Out of 150 patients with COVID-19, the majority (60) of the patients were in the group of 41-60 years. Diabetes mellitus (36) and hypertension (29) were the most common comorbidities found in this study. All other baseline characteristics of COVID-19 among survivors and non-survivors have been highlighted in Table 1.

**Table 1:** Comparison of base parameters S: statistically significant; NS: statistically not significant

Parameters	Survivors (N = 102)	Non-survivors (N = 48)	P-value
Age (years)			
≤20	2 (1.96%)	0 (0%)	0.155 NS
21-40	27 (26.47%)	6 (12.5%)	0.054 NS
41-60	41 (40.19%)	19 (39.58%)	0.943 NS
>60	32 (31.37%)	23 (47.92%)	0.050 NS
Mean ± SD	52.14 ± 15.00	60.50 ± 15.73	0.002 S
Gender			
Male	74 (72.55%)	35 (72.92%)	0.962 NS
Female	28 (27.45%)	13 (27.08%)	
Comorbidities			
Diabetes	11 (10.78%)	18 (37.5%)	<0.001 S
Hypertension	16 (15.69%)	20 (41.67%)	0.001 S
Asthma	3 (2.94%)	11 (22.92%)	<0.001 S
Adverse habits			
Alcohol	4 (3.92%)	8 (16.67%)	0.007 S
Smoking	6 (5.88%)	10 (20.83%)	0.001 S
Disease severity			
Mild	44 (43.14%)	3 (6.25%)	<0.001 S
Moderate	26 (25.49%)	20 (41.67%)	0.045 S
Severe	32 (31.37%)	25 (52.08%)	0.015 S

Among the biochemical parameters, leukocyte count, PLT, and inflammatory markers were found to be significant in survivors and non-survivors of COVID-19 as shown in Table 2.

**Table 2:** Comparison of other parameters among survivors and non-survivors S: statistically significant: NS: statistically not significant; IL-6: interleukin-6; LDH: lactate dehydrogenase; CRP: C-reactive protein; MPV: mean platelet volume; PCT: plateletcrit; PDW: platelet distribution width; PLT: platelet count; FEU: fibrinogen equivalent units

Parameters	Survivors (N = 102)	Non-survivors (N = 48)	P-value
Serum creatinine (mg/dL) (mean ± SD)			
Hemoglobin (gm/dL)	12.47 ± 2.39	12.34 ± 2.37	0.761 NS
Leukocyte count (/cu. mm)	7294.12 ± 2471.25	10495.83 ± 3712.71	<0.001 S
PLT (lakh/cu. mm)	2.03 ± 0.72	1.76 ± 0.47	0.018 S
Serum creatinine (mg/dL)			
Serum creatinine (mg/dL)	1.15 ± 0.68	1.57 ± 0.86	0.001 S
Serum electrolytes			
Sodium (mEq/L)	140.26 ± 4.86	139.98 ± 5.33	0.745 NS
Potassium (mEq/L)	4.51 ± 0.66	4.72 ± 0.89	0.105 NS
Inflammatory markers			
IL-6	125.89 ± 145.56	230.08 ± 110.10	<0.001 S
D-dimer (mg/L FEU)	2.34 ± 1.81	5.56 ± 1.94	<0.001 S
Ferritin (ng/mL)	316.51 ± 123.75	609.69 ± 333.59	<0.001 S
LDH (U/L)	357.30 ± 120.86	743.67 ± 339.27	<0.001 S
CRP (mg/L)	3.64 ± 2.51	12.68 ± 4.14	<0.001 S
Platelet indices			
MPV (µ cu. mm)	9.22 ± 0.64	10.42 ± 0.53	<0.001 S
PCT (%)	0.22 ± 0.03	0.18 ± 0.33	<0.001 S
PDW (fl)	16.54 ± 0.91	17.99 ± 1.53	<0.001 S

Table 3 depicts the comparisons of disease severity among both survivors and non-survivors. The mean PLT was significantly higher in survivors than non-survivors who had moderate (41.67% versus 25.49%; p-value = 0.045) and severe COVID-19 (52.08% versus 31.37%; p-value = 0.015). However, a significantly greater proportion of survivors than non-survivors had mild COVID-19 (43.14% versus 6.25%; p-value <0.0001). 

**Table 3:** Comparisons of disease severity among both survivors and non-survivors

Disease severity	Survivors (N = 102)	Non-survivors (N = 48)	P-value
Mild	44 (43.14%)	3 (6.25%)	<0.001
Moderate	26 (25.49%)	20 (41.67%)	0.045
Severe	32 (31.37%)	25 (52.08%)	0.015

Table 4 depicts the comparisons of platelet indices among survivors than non-survivors. The mean PLT was noticeably greater among survivors than non-survivors (2.03 ± 0.72 lakh/cu. mm versus 1.76 ± 0.47 lakh/cu. mm; p-value = 0.018). The mean MPV (10.42 ± 0.53 µ cu. mm versus 9.22 ± 0.64 µ cu. mm; p-value <0.0001) and PDW (17.99 ± 1.53 fl versus 16.54 ± 0.91 fl; p-value <0.0001) were somewhat greater among non-survivors than survivors. However, the presumed PCT was much higher in survivors than in non-survivors (0.22 ± 0.03% versus 0.18 ± 0.33%; p-value <0.0001).

**Table 4:** Comparisons of platelet indices among both survivors and non-survivors PLT: platelet count; MPV: mean platelet volume; PCT: plateletcrit; PDW: platelet distribution width

Platelet indices	Survivors (N = 102)	Non-survivors (N = 48)	P-value
PLT (lakh/cu. mm)	2.03 ± 0.72	1.76 ± 0.47	0.018
MPV (µ cu. mm)	9.22 ± 0.64	10.42 ± 0.53	<0.001
PCT (%)	0.22 ± 0.03	0.18 ± 0.33	<0.001
PDW (fl)	16.54 ± 0.91	17.99 ± 1.53	<0.001

Across the severity groups, the mean MPV varied considerably (p-value = 0.001). According to a post hoc study, individuals with severe COVID-19 had presumed MPV values that were considerably higher than those with moderate COVID-19 (p-value = 0.001). Nevertheless, no statistically significant change in the mean PCT or mean PDW was found across the severity groups (p-values = 0.227 and 0.327, respectively) as shown in Table 5.

**Table 5:** Results are compared of the mean MPV (mean ± SD), PCT (%), and PDW (fl) based on the severity of COVID-19 S: statistically significant: NS: statistically not significant; MPV: mean platelet volume; PCT: plateletcrit; PDW: platelet distribution width

Parameters	Mild (N = 47)	Moderate (N = 46)	Severe (N = 57)	P-value
MPV (µ cu. mm)	9.27 ± 0.61	9.64 ± 0.92	9.85 ± 0.82	0.001 S
PCT (%)	0.22 ± 0.03	0.20 ± 0.04	0.20 ± 0.04	0.227 NS
PDW (fl)	16.79 ± 0.95	17.21 ± 1.59	17.01 ± 1.36	0.327 NS

Multiple regression analysis for MPV was significantly associated with diabetes mellitus (p-value = 0.003), hypertension (p-value = 0.005), and asthma (p-value = 0.012) as shown in Table 6.

**Table 6:** Analysis of multiple regression for MPV MPV: mean platelet volume

Pattern		Unreliable coefficients		Normative coefficients	t	P-value
		B	Std. error	Beta		
1	(Persistent)	9.377	.271		34.548	.000
	Age	-.051	.134	-.030	-.381	0.704
	Sex	.012	.147	.006	.080	0.936
	Diabetes mellitus	.510	.167	.245	3.047	0.003
	Hypertension	.435	.152	.226	2.859	0.005
	Asthma	.579	.228	.205	2.541	0.012
	Alcohol	.170	.249	.056	.685	0.494
	Smoking	.139	.213	.052	.654	0.514

Multiple regression analysis for PCT was significantly associated with male sex (p-value = 0. 025) and asthma (p-value <0.0001) as shown in Table 7.

**Table 7:** Multiple regression analysis for PCT PCT: plateletcrit

Pattern		Unreliable coefficients		Normative coefficients	t	Sig.
		B	Std. error	Beta		
1	(Persistent)	.252	.012		20.614	.000
	Age	-.012	.006	-.152	-1.942	0.054
	Sex	-.015	.007	-.177	-2.269	0.025
	Diabetes mellitus	-.012	.008	-.127	-1.615	0.108
	Hypertension	-.008	.007	-.090	-1.165	0.246
	Asthma	-.040	.010	-.311	-3.934	.000
	Alcohol	-.016	.011	-.112	-1.394	0.166
	Smoking	.004	.010	.029	.372	0.711

Multiple regression analysis for PDW was significantly associated with age ≥60 years (p-value = 0.043), male sex (p-value = 0.046), diabetes mellitus (p-value = 0.020), and asthma (p-value = 0.024) as shown in Table 8.

**Table 8:** Multiple regression analysis for PDW PDW: platelet distribution width

Pattern		Unreliable coefficients		Normative coefficients	t	Sig.
		B	Std. error	Beta		
1	(Persistent)	15.452	.436		35.423	.000
	Age	.440	.215	.163	2.043	0.043
	Sex	.475	.236	.160	2.015	0.046
	Diabetes mellitus	.633	.269	.189	2.355	0.020
	Hypertension	.236	.244	.076	.964	0.337
	Asthma	.834	.366	.183	2.277	0.024
	Alcohol	.524	.400	.107	1.312	0.192
	Smoking	.276	.342	.064	.807	0.421

**Figure 1:**
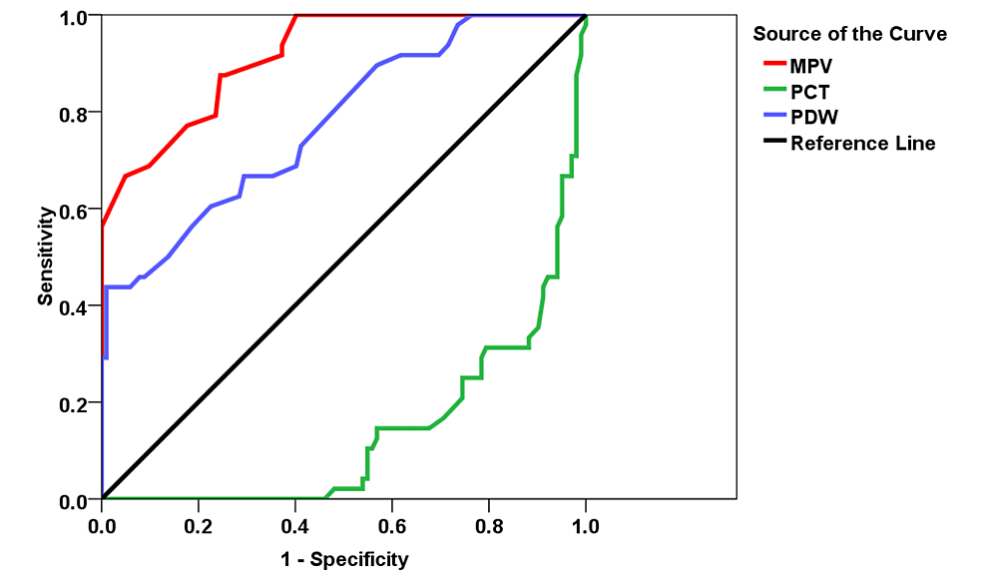
ROC curve and AUC for predicting deaths ROC: receiver operating characteristic; AUC: area under curve; MPV: mean platelet volume; PCT: plateletcrit; PDW: platelet distribution width

Regarding the ROC curves for MPV in predicting death having a cut-off of 9.75, the sensitivity and specificity of MPV in predicting death were found to be 87.5% and 75.5%. As regards the ROC curves for PCT in predicting death having a cut-off of 0.213, the sensitivity and specificity in predicting mortality were discovered to be 79.2% and 74.5%, respectively. The area under the curve (AUC) and p-values were 0.777 (95% confidence interval (CI): 0.697-0.857) and <0.0001, respectively. With regard to the ROC curves for PDW in predicting mortality having a cut-off of 16.75, the sensitivity and specificity of PDW in predicting death were found to be 68.8% and 59.8%, respectively, as shown in Figure 1.

## Discussion

The clinical course of COVID-19 can quickly deteriorate to fatal consequences and ranges in severity from asymptomatic to a serious systemic illness with multiorgan and/or respiratory involvement [17-21]. White blood cell counts and MPV are utilized as indicators of the inflammatory response as a reflection of prothrombotic and pro-inflammatory factors such as a variety of cytokines of inflammation, including IL-1, IL-6, and TNF, which influences thermophoresis [21,22]. It has been demonstrated that the size of the circulating platelets may be used to categorize diseases based on the degree of systemic inflammation [23]. Sepsis has been associated with greater MPV and greater PDW, and PDW has been demonstrated to be a poor predictor in the outcome of severe sepsis. Compared to patients without COVID-19, MPV is considerably higher in COVID-19 patients [24]. Preliminary research has suggested that an increase in MPV and PDW is linked to increased mortality as a result of COVID-19 [24].

Age has been identified as the primary factor influencing prognosis in COVID-19 patients since the beginning of the epidemic. According to early Chinese statistics data, the case fatality rate (CFR) rises sharply after age 60 and reaches 14.8% for people over the age of 80 [25]. According to preliminary statistics from Italian patients, mortality rose noticeably in patients in their 70s and nearly quadrupled in the 80+-year-old. Age was discovered to be a reliable predictor of death in Chinese cohort research and has a 1.10 incidence (95% self-belief range 1.03, 1.17) for each year [26]. In the current study, the age ranges of the majority of survivors and non-survivors were 41-60 years (40.19%) and >60 years (47.92%), respectively. The non-survivors' mean age was noticeably older than the survivors. Like the current study, Asghar et al. found that although there was no discernible difference, compared to non-survivors, survivors had a greater average age [24].

As cases spread over the world, it has been noted that people with underlying chronic diseases are more likely to contract the virus and become seriously ill. In contrast to those without comorbidities, patients with comorbidities experience higher adverse outcomes. Those with COVID-19 who have a history of cardiovascular illness, diabetes, obesity, chronic obstructive pulmonary disease, or hypertension have the poorest prognosis and are more likely to experience worsening outcomes including ARDS and pneumonia. In addition, people with chronic renal illness and cancer and the elderly in long-term care institutions not only are in danger of catching the virus but also have a considerably higher chance of dying from it [27].

Asthma, diabetes, and hypertension were considerably less frequent in survivors than in non-survivors in the current research. Similar to this study, Ge et al. revealed that diabetes and hypertension had increased mortality. In people with asthma taking medium and high dosages of inhaled corticosteroids (ICS), Talwar et al. found an increased incidence of COVID-19 mortality [28]. Moreover, Han et al. [29] and Hou et al. [30] revealed in their meta-analysis that asthma was linked to considerably lower COVID-19-related risk mortality. As the outcome, COVID-19 individuals with comorbidities have a higher chance of mortality.

Early on in the COVID-19 infection, fast viral replication and the production of powerful pro-inflammatory cytokines cause primary inflammation. Together with extensive alveolar injury and pulmonary infiltration, widespread endothelial inflammation brought on by a viral infection of endothelial cells might promote the release of more inflammatory cytokines [31,32]. Inflammation and illness severity has been associated with COVID-19. It is reported that cytokine storm is the cause of severe multisystemic end-organ failure-associated mortality. CRP is discovered to be causally connected to greater COVID-19 danger. In the beginning, the severity of the illness is correlated with CRP levels. The likelihood of developing severe COVID-19 and higher mortality are both associated with high LDH levels [33,34].

In the current study, incapables had substantially stronger mean levels of IL-6, D-dimer, ferritin, LDH, and CRP than survivors. Moreover, a significantly greater proportion of non-survivors than survivors had moderate and severe COVID-19. Similar to the present study, Bawiskar et al. observed that higher levels of the poorer results were linked to higher levels of inflammatory markers such as ferritin, CRP, IL-6, procalcitonin, and lactic acid [35]. Moreover, non-survivors exhibited considerably higher mean D-dimer, ferritin, and CRP levels than survivors, according to Asghar et al. However, mean LDH levels did not differ significantly [24,36]. Another study by Walinjkar et al. revealed that the percentage of non-survivors with mean CRP levels was much greater than the percentage of survivors [22]. According to Lazar et al., ferritin, CRP, and IL-6 did not substantially vary from non-survivors, although mean LDH and D-dimer were considerably elevated [36,37]. Individuals with severe COVID-19 had increased mortality and elevated inflammatory markers.

In addition to aiding in haemostasis and coagulation, platelets are crucial for innate immunity and the inflammatory response [38,39]. According to multiple research, inflammatory and prothrombotic reactions to a variety of viral infections affect platelet indices [40,41]. Cytokines stimulate platelets, which are crucial in inflammation.

The mean MPV varied considerably among the severity groups in the current research. According to a post hoc analysis, individuals with severe COVID-19 illness had mean MPV values that were considerably greater than those with milder disease. Nevertheless, there was no discernible difference in the mean PCT or PDW across the severity groups. Similar findings were made by Quispe-Pari et al. who discovered that those with severe COVID-19 had considerably higher mean MPV [42]. Further research by Yardimci et al. [5] and Walinjkar et al. [22] showed that PDW did not significantly correlate with the severity of the condition.

Mean MPV and PDW in the current research were substantially higher among non-survivors than survivors. Yet, among survivors compared to non-survivors, the mean PCT was substantially higher [22]. Another study indicated that the mean of non-survivors was substantially higher in MPV and PDW, while PCT did not differ between survivors and non-survivors [33]. According to Gupta et al., non-survivors had MPV that was significantly greater than survivors [43]. A substantial association between MPV and Acute Physiology and Chronic Health Evaluation (APACHE) score, a grading system, indicated that the mean of non-survivors was substantially higher. Death was also discovered by Kucukardali et al. [44,45]. Hence, greater MPV and PDW levels are associated with an increased risk of passing away from COVID-19-related causes.

In the present study, at a reduction of 9.75, the personalization and responsiveness of MPV in predicting death were found to be 87.5% and 75.5%, respectively (AUC: 0.915; p-mean <0.0001). Related to the current investigation, Dubey et al. discovered that MPV was the most crucial factor and that death was predicted by values more than 10.05 with a sensitivity and specificity of 69.5% and 69.3%, respectively [46]. In a different research, Quispe-Pari et al. used a cut-off of 10.15 and showed a 59% sensitivity and a 14% specificity in forecasting death [42,47]. According to Shah et al., MPV exhibited a sensitivity and precision of 66.3% and 62.8%, respectively, in predicting mortality at a cut-off of 10.45 [48,49]. Thus, the prognostic value of MPV in predicting morality is identical to that observed in the literature.

Also, we found that PDW had a sensitivity and specificity of 68.8% and 59.8% in predicting mortality, respectively, at a cut-off of 16.75. Kumar et al. considered a cut-off of 17 and reported a sensitivity of 84% and specificity of 44% in predicting mortality [50,51]. These findings concur with those made public by Yun et al. [47,51] and Walinjkar et al. [22].

The PCT's predictive accuracy and precision mortality are found to be 79.2% and 74.5%, respectively, in the current study when a cut-off of 0.213 was taken into account. To the best of our knowledge, PCT's prognostic usefulness in predicting death is not yet identified in COVID-19 patients [52].

Limitation

This study involved a relatively small number of patients admitted in a single centre; hence, the results cannot be generalized to the community. The survivors were not followed up, and thus, the prognoses of these patients remain unknown. Abnormalities in platelet functions were not evaluated.

## Conclusions

The study came to the conclusion that platelet indices like higher MPV and PDW are linked to severe COVID-19 and mortality, but higher PCT is linked to a better prognosis. Platelet indices easily obtained from a complete hemogram are frequently used in every healthcare centre and can be utilized as low-cost, prognostic indicators in these patients. Moreover, there may be a less need of performing a lot of expensive serological (D-dimer assay, CRP, or IL-6) and radiological (chest HRCT) investigations, which may be not accessible for populations of all income levels.
